# Supplementary material for: An automatic multi-tissue human fetal brain segmentation benchmark using the Fetal Tissue Annotation Dataset
Source: Sci Data. 2021 Jul 6;8:167. doi: 10.1038/s41597-021-00946-3 (PMC8260784; doi:10.1038/s41597-021-00946-3)
Supplement: Supplementary file 1 — Supplemental Information [file 41597_2021_946_MOESM1_ESM.pdf]

# Supplemental Information

## Table of Contents

Supplemental Information 1

**1. Overview of each algorithm by label 2**
1.1 Multi-Atlas Segmentation 2
1.2 SeBRe 2
1.3 IBBM Method 3
1.3.1 Axial Orientation 3
1.3.2 Sagittal Orientation 3
1.3.3 Coronal Orientation 4
1.3.4 Combined 4
1.4 Kispi Method 5
1.4.1 2D axial orientation 5
1.4.2 2D sagittal orientation 5
1.4.3 2D coronal orientation 6
1.4.4 3D orientation 6

**2. Fetal Brain Annotation Guideline 7**

# 1. Overview of each algorithm by label

In the following sections, the evaluation metrics for each algorithm separated by label number are displayed. (CSF: external cerebrospinal fluid; GM: Grey Matter; WM: White Matter)

## 1.1 Multi-Atlas Segmentation

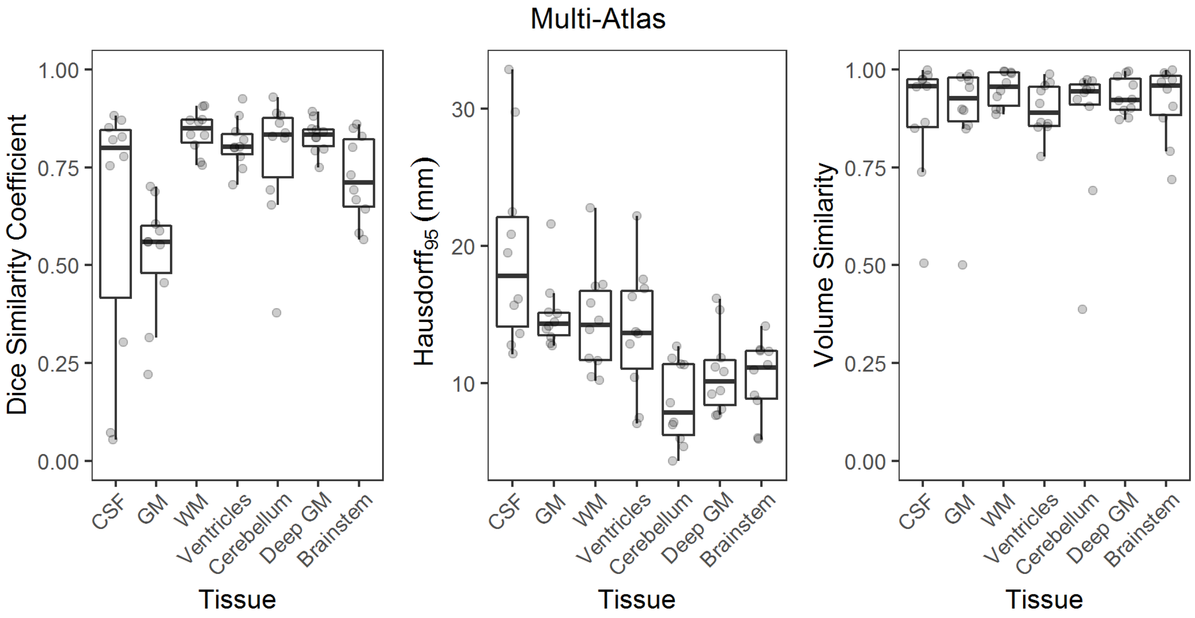


## 1.2 SeBRe

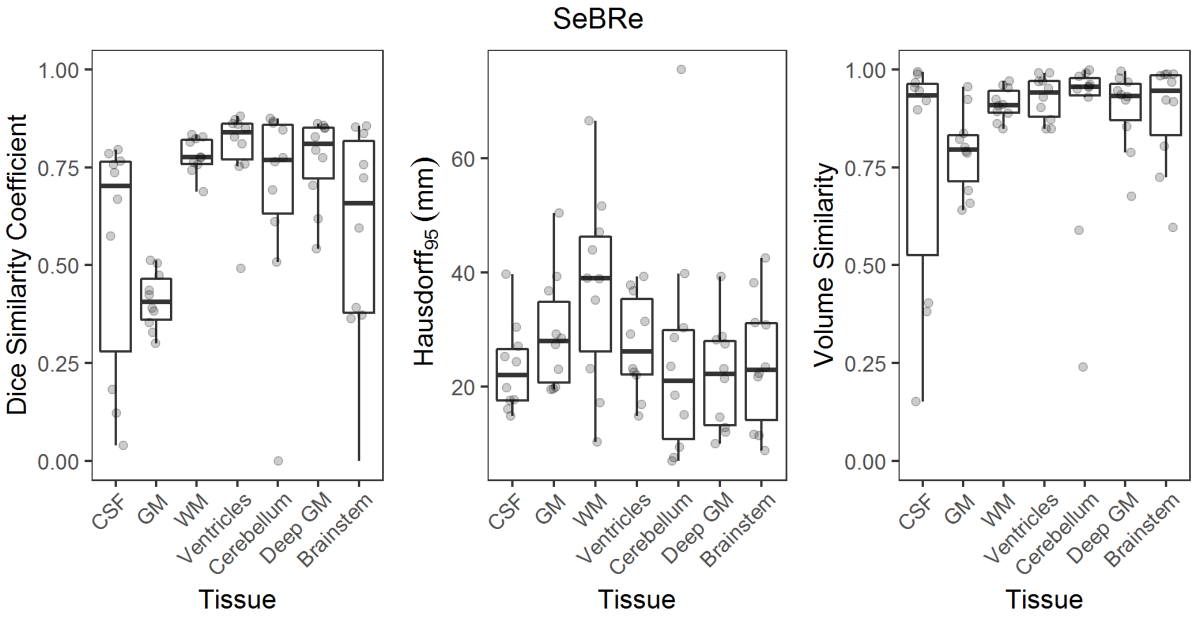

## 1.3 IBBM Method

### 1.3.1 Axial Orientation

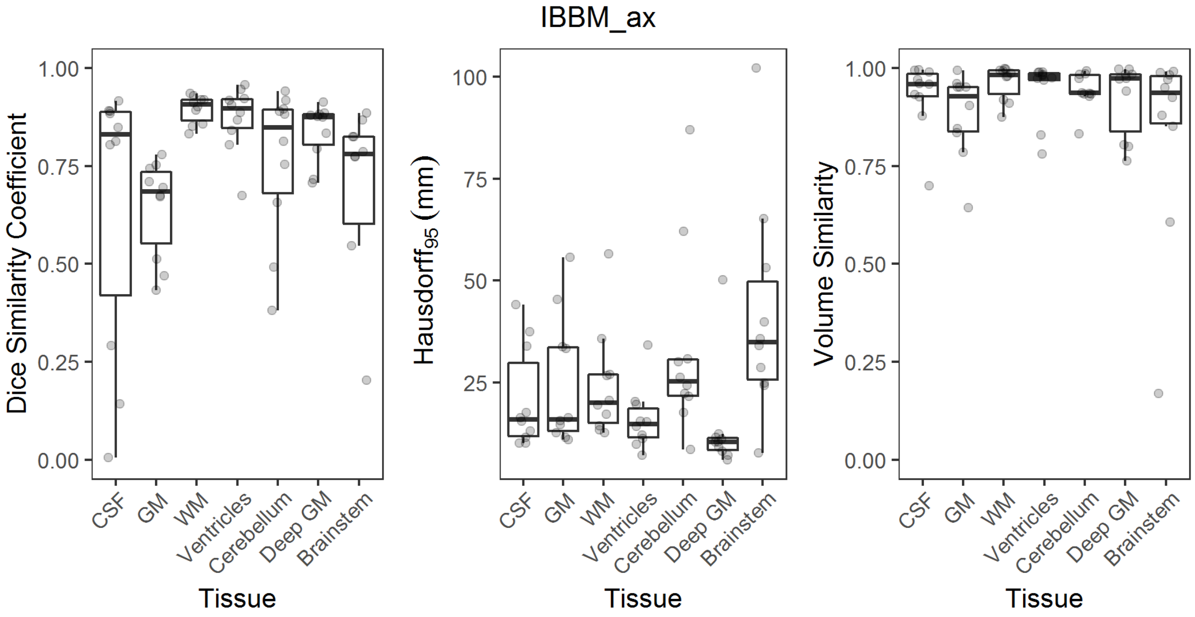


### 1.3.2 Sagittal Orientation

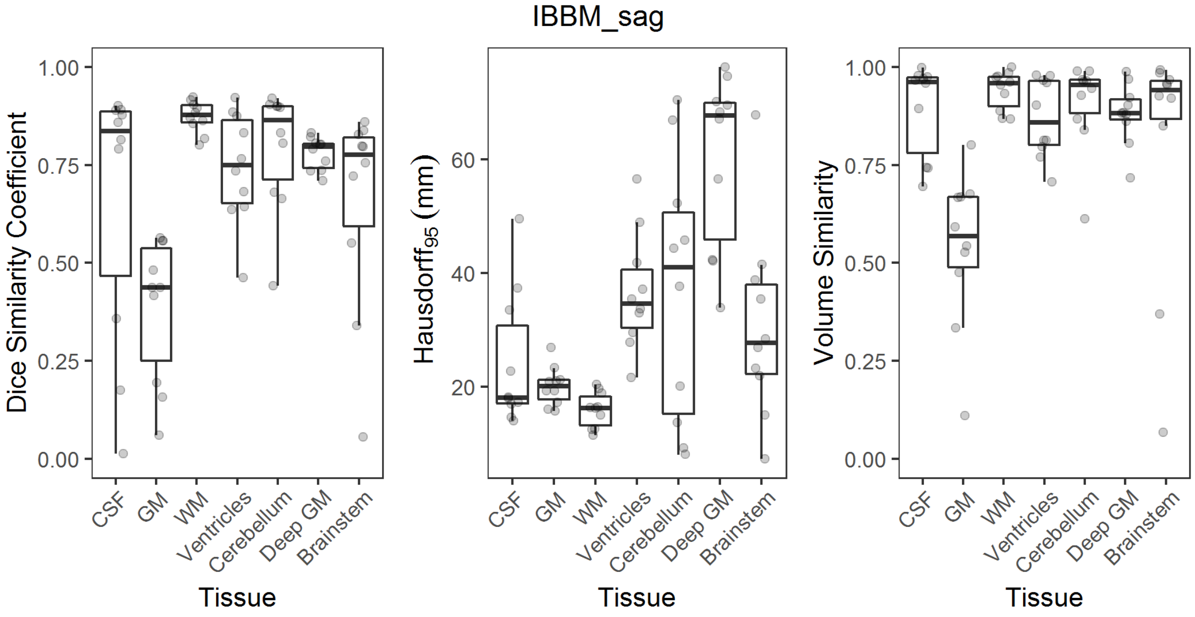

### 1.3.3 Coronal Orientation

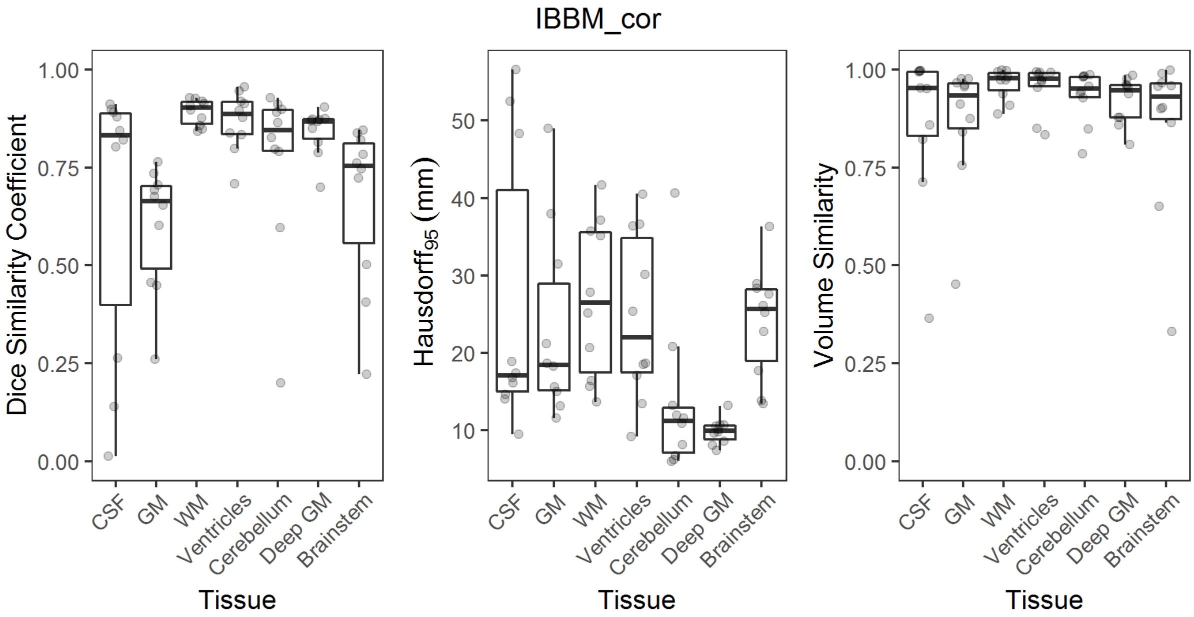


### 1.3.4 Combined

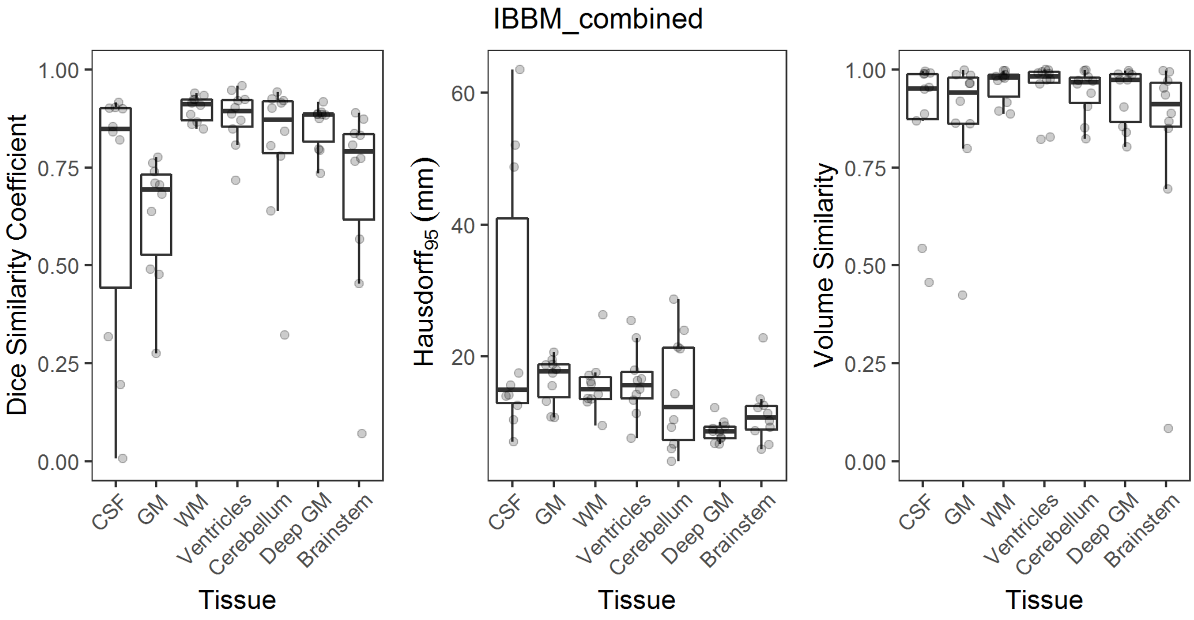

## 1.4 Kispi Method

### 1.4.1 2D axial orientation

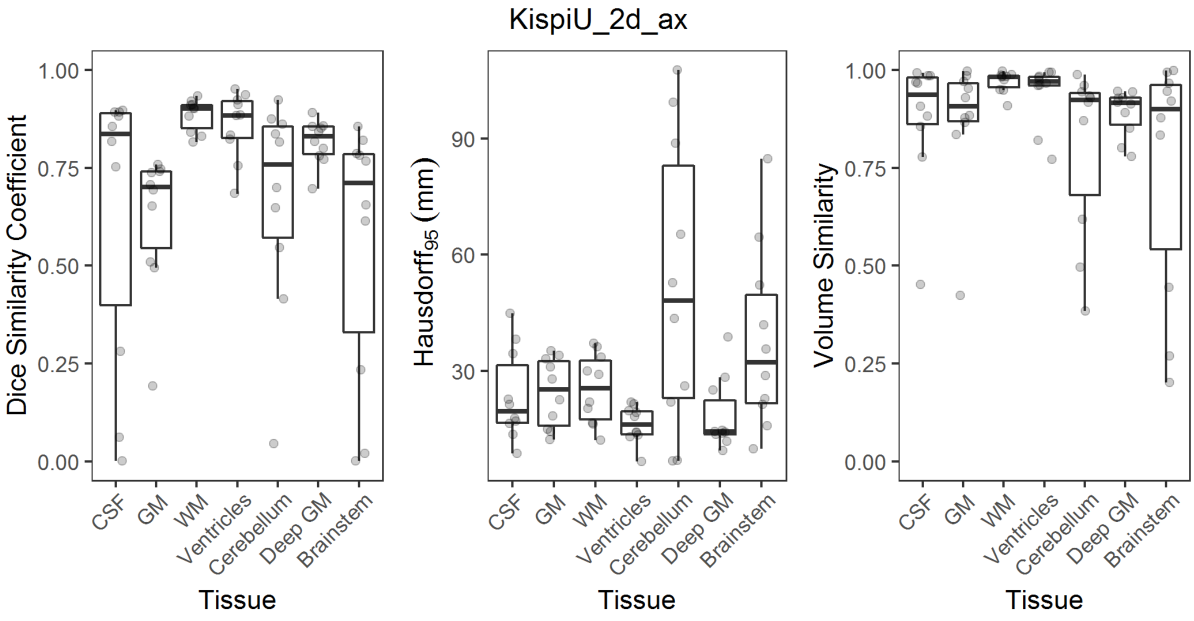


### 1.4.2 2D sagittal orientation

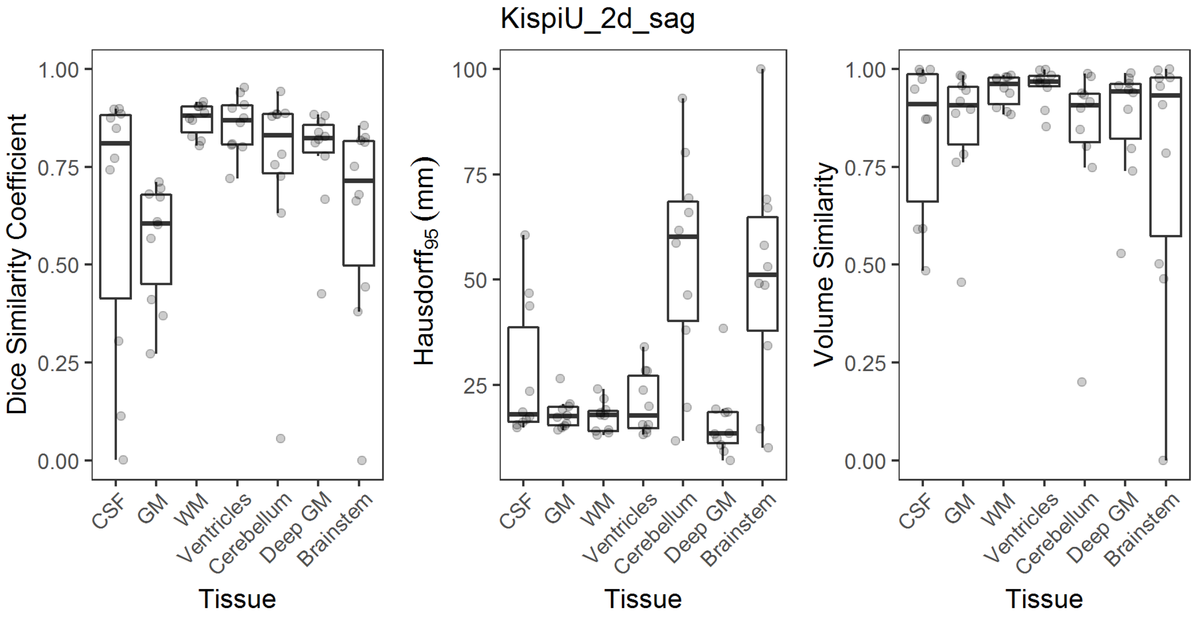

### 1.4.3 2D coronal orientation

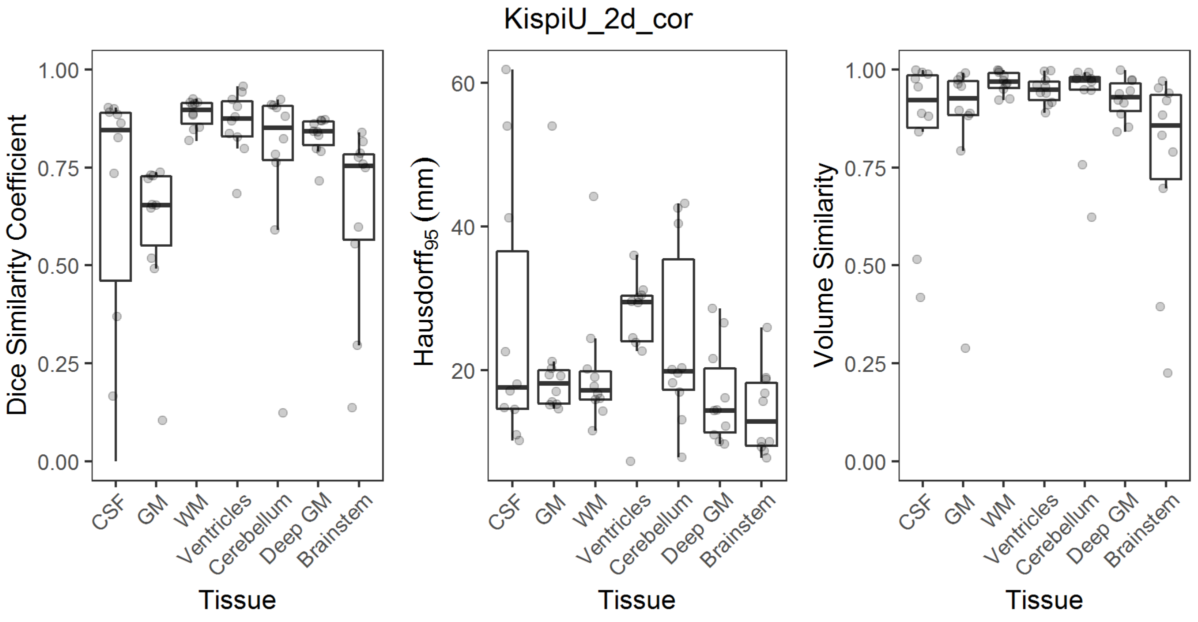


### 1.4.4 3D orientation

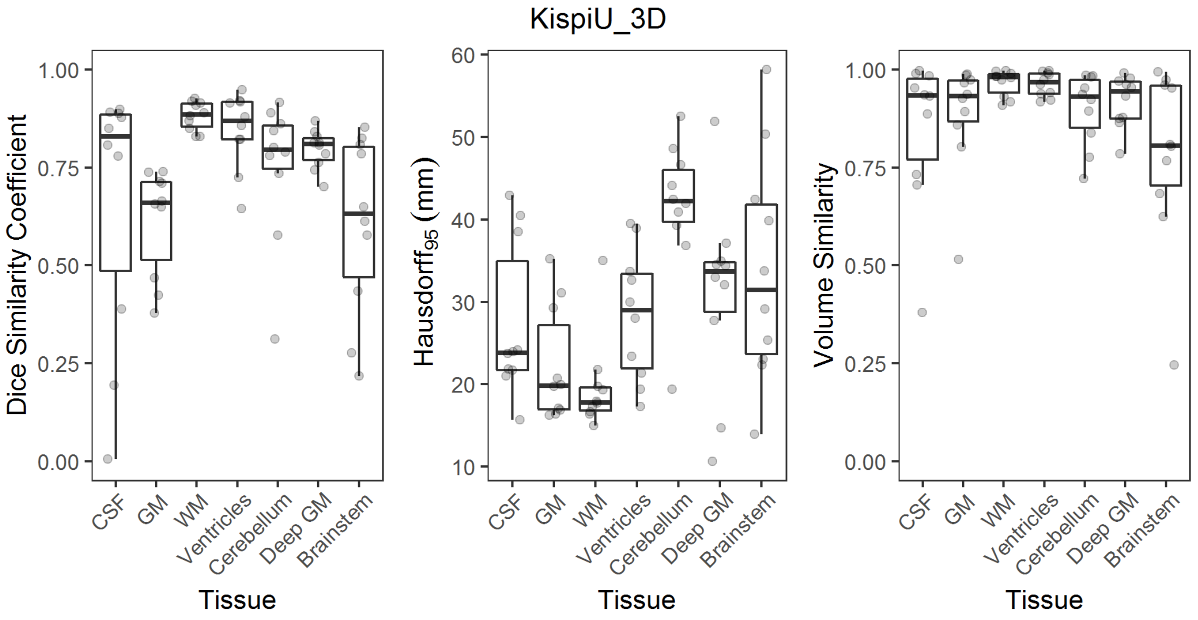

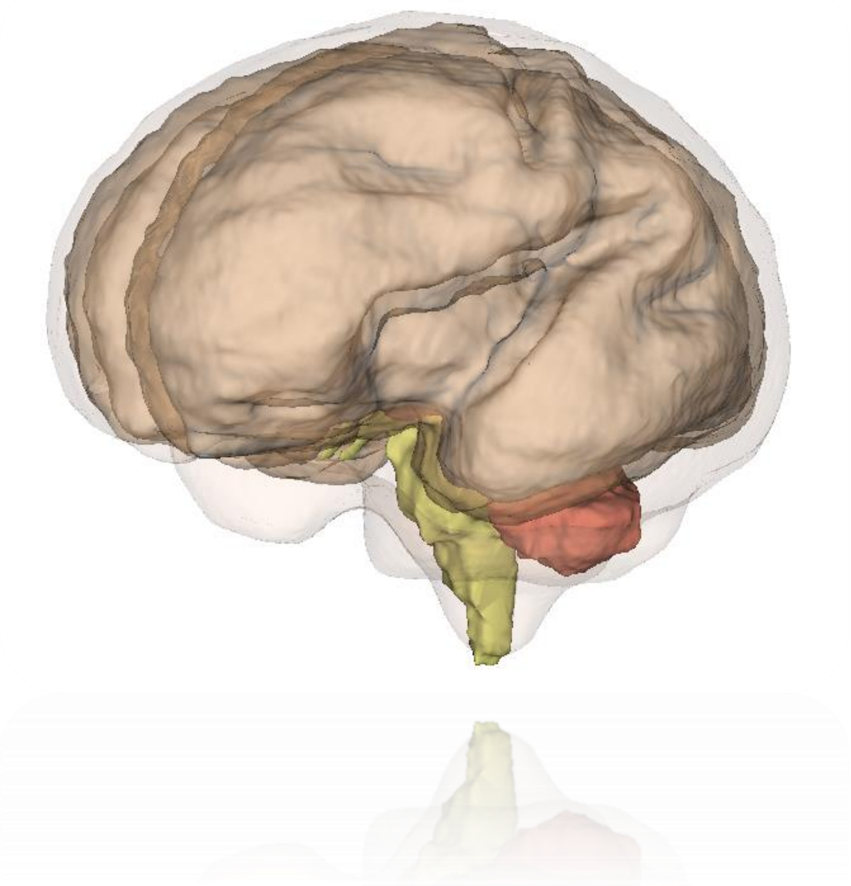

# Fetal brain annotation guideline

V. 1.3 8/4/2020

© Andras Jakab, University of Zurich

This is a guideline for providing manual expert annotations for fetal super-resolution T2-weighted MRI data. The purpose of this document is to provide technical and anatomical guidance for annotators for the fetal neuroimaging projects at the University of Zürich, or for other groups. The labeling scheme and definitions are based on the **dHCP pipeline** for neonatal data sets (*Makropoulos et al. 2018*). The manual expert annotations are post processed and combined into a final label map. The combined label maps comprise the following labels:

| dHCP label | Name | Notes |
|---|---|---|
| Label 1 | Intracranial space and extra-axial CSF spaces | - |
| Label 2 | Gray matter | - |
| Label 3 | White matter | - |
| Label 4 | Ventricles | - |
| Label 5 | Cerebellum | - |
| Label 6 | Deep gray matter | - |
| Label 7 | Brainstem and spinal cord | - |

The recommended software is Slicer 3D, version 4 and above. Please open the 3D T2 volume, and let Slicer create a new label map for each structure **separately** when using the **Editor** module (Master volume: T2, Merge volume: Create new LabalMapVolume). The naming of the label maps or label map image files is not important, however, it is crucial that the given label number is used for annotation (for example, gray matter: 2), and that only one label is saved in each label map file. Otherwise the automated label combination script will not work. After the annotation of one label map has been completed, please save it in a **folder separately** for each patient, and select **.nii.gz** format in the Save module of Slicer.

## Intracranial space / fluid background (label: 1)

**Annotation plane:** axial
**Annotation style:** manual annotation on every 5$^{th}$ slice

**Description:** this is a label for the fetal intra-cranial space or external fluid spaces. The annotator has to draw a round outline around the outer CSF spaces. It is important to exclude the eyes and any non-CSF, non-intra-axial structures, such as parts of the amniotic fluid from which parts appear outside to the skull (**Figure 1**).

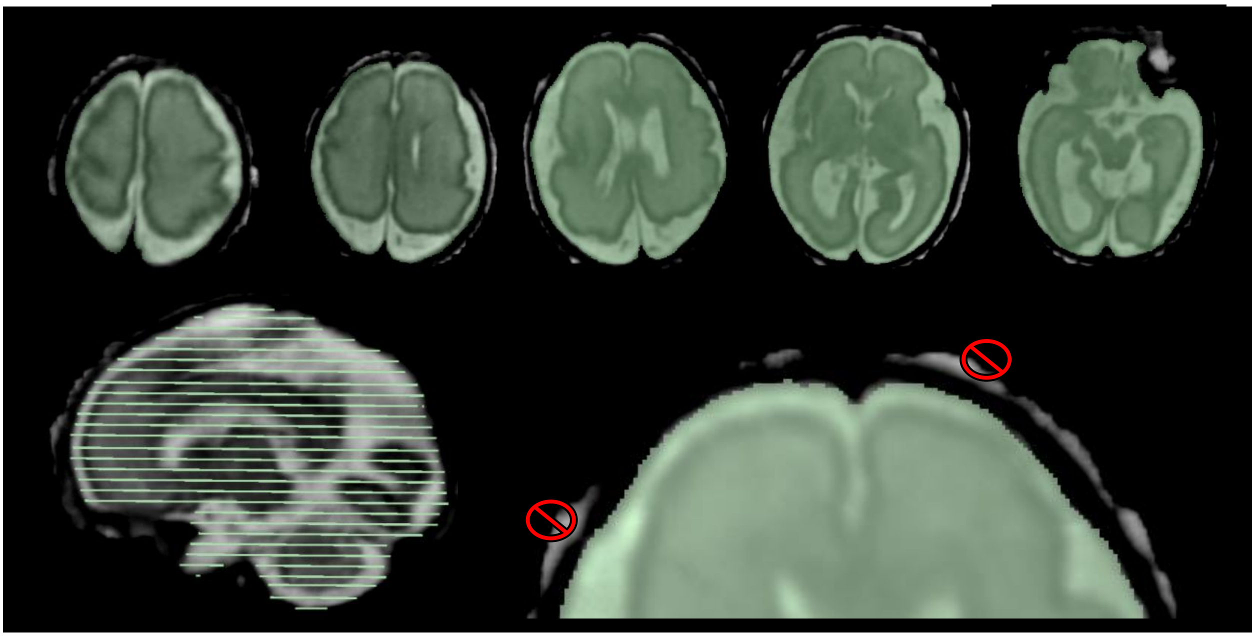

***Figure 1:*** *Label 1: annotating the fluid background (intra-cranial space of the fetal brain, label: green). Top row: axial cross-sections, bottom row: sagittal image.*

## <u>External border of the cortex cerebri</u> (label: 2)

**Annotation plane:** axial
**Annotation style:** manual annotation on every 2nd slice on the top of 10 slices of the brain, every 3rd slice elsewhere, i.e. in the middle section of the brain.

**Description:** this is a label for the external brain surface and cortex, only supratentorially. This label will be combined with the white matter and subcortical labels, therefore it is important to make sure that it is not a hollow label. The thalamus / mesencephalon region can be included in this label map, as it will be overwritten. This label has to be precise and each sulcus and gyrus should be outlined with high accuracy (see **Figure 2**). The label includes the amygdala and hippocampus, but does not include any infratentorial structures.

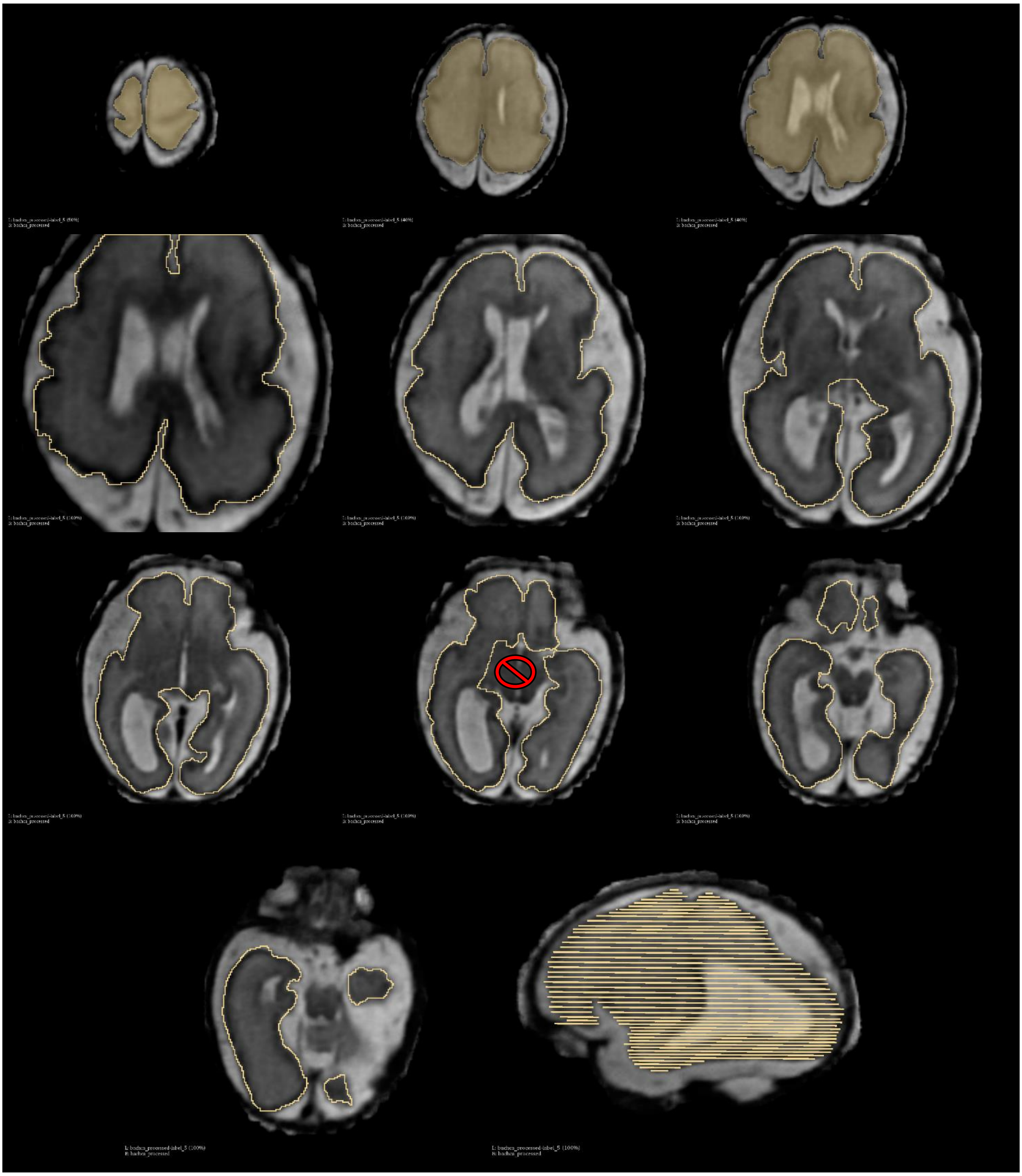

***Figure 2:*** *Label 2: the external border of the cortex. Red sign: regions, such as deep gray matter, brainstem and cerebellum will be taken from other annotations, therefore it is not important to precisely exclude them from the label map, and rough "internal" borders are sufficient.*

When it is difficult to distinguish between the corpus callosum, cingular/other cortex, these should be included in the label map, as shown in **Figure 3**:

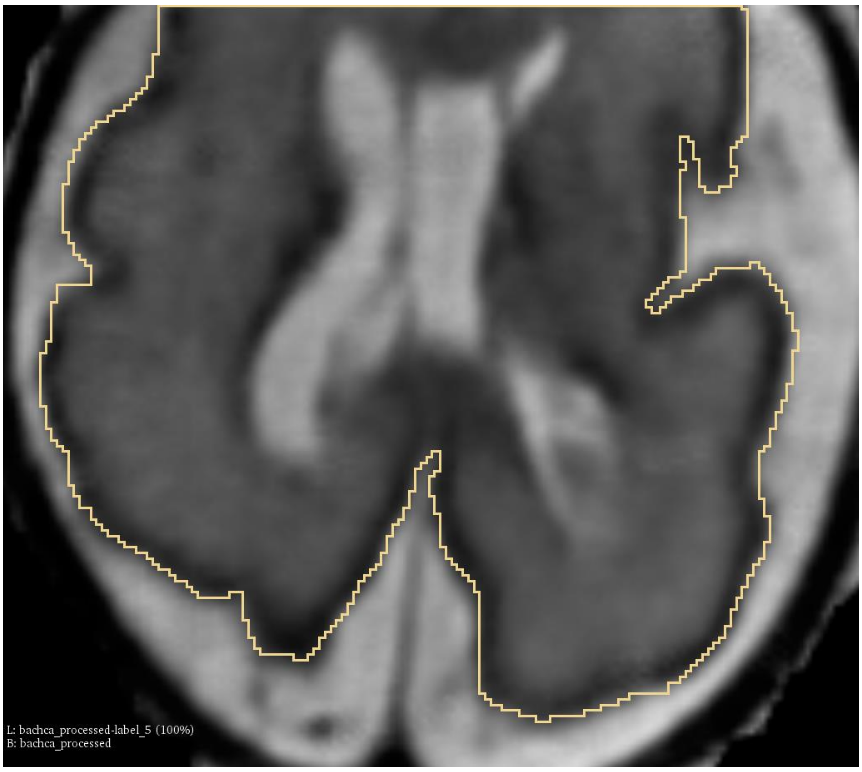


***Figure 3:*** *Label 2: the external border of the cortex, region of the corpus callosum. When in doubt, include non-gray matter regions in the label, such as the corpus callosum.*

Please include the lateral sulcus and pay attention to the CSF spaces in the lateral sulcus, as shown in **Figure 4**.

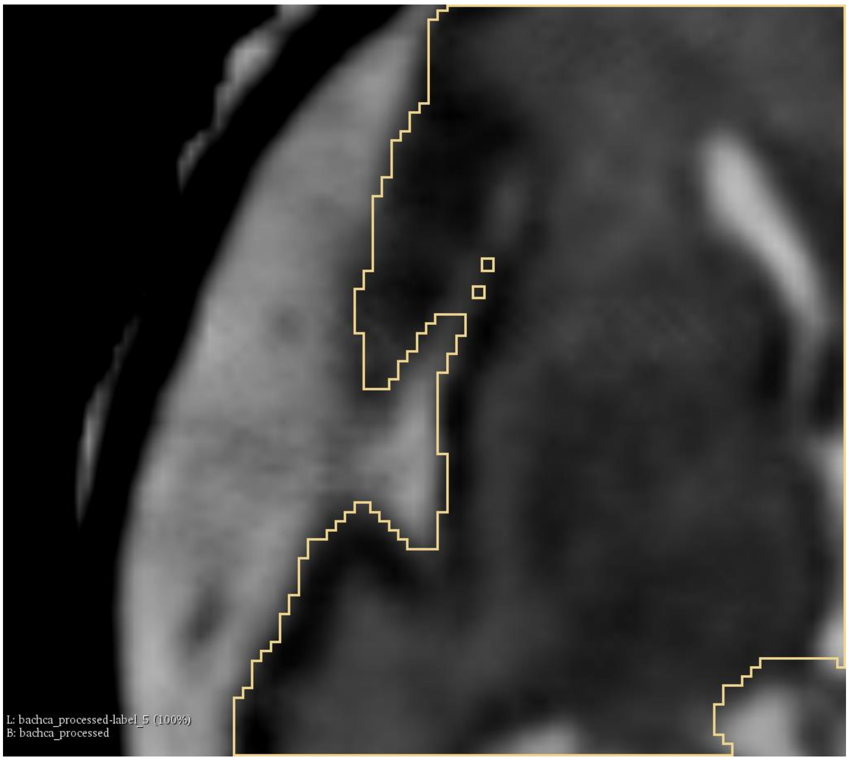


***Figure 4:*** *Label 2: the external border of the cortex, region of the lateral. If possible, please follow the borders in the sulci as well, for example, in the lateral sulcus around the insula.*

## External border of the white matter (label: 3)

**Annotation plane:** axial
**Annotation style:** manual annotation on every 2nd slice on the top of 10 slices of the brain, every 3rd slice elsewhere

**Description:** this is a label for the external border of the supratentorial white matter, which also encloses all structures that are more central to the white matter. This label will be combined with all labels of higher numbers (subcortex, ventricles, etc.), therefore the annotator has to include all of these structures as well, similarly to the cortex. The cortex / white matter interface has to be annotated precisely. Whenever possible, the white matter should be separated from the cortex, based on anatomical knowledge (**Figure 5**):

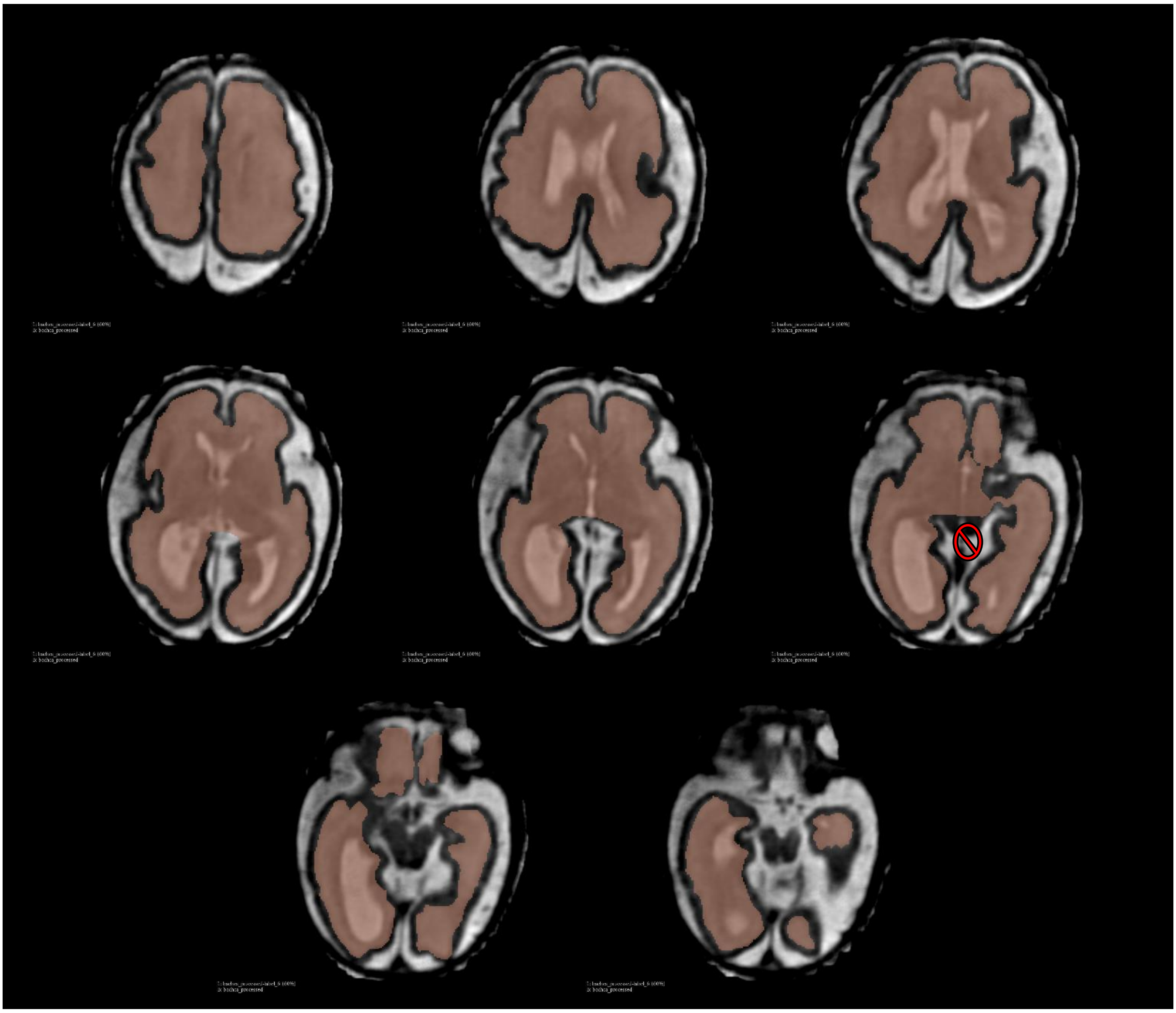

***Figure 5:*** *Label 3: the external border of the supratentorial white matter. Red "no" sign: regions, such as deep gray matter, brainstem and cerebellum will be taken from other annotations, therefore it is not important to precisely exclude them from the label map. Less precise "internal" borders are sufficient.*

The region where the two cerebral hemispheres lie closely can be annotated (**Figure 6/a**). Similarly to the gray matter label, it is not necessary to exclude non-white matter structures, it is only important that the OUTER borders are respected and non-brain tissues are excluded (**Figure 6/b**). Make sure that the white matter is always covered by cortex, even if the signal is bad and the cortex appears whiter than usual (**Figure 6/c**).

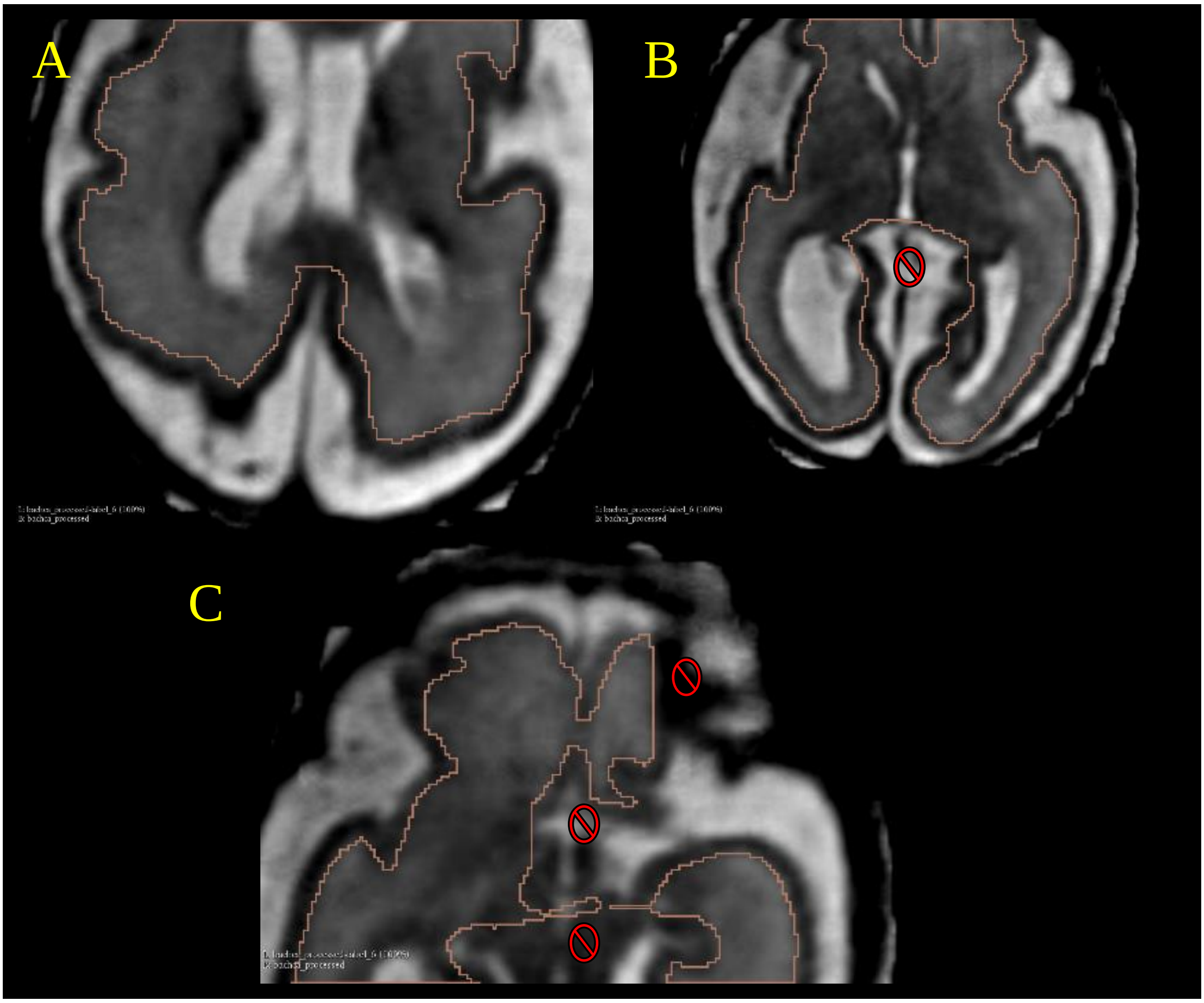


***Figure 6:*** *Label 3: the external border of the supratentorial white matter. a: region of the corpus callosum, b: inclusion of non-white matter tissues in the label. C: segmentation of parts (e.g. entorhinal cortex) with larger imaging artifacts. NO sign: parts that are not important to be segmented precisely in the label, as they will be overwritten by other labels, such as the brainstem.*

## <u>Cerebral ventricles</u> (label: 4)

**Annotation plane:** axial
**Annotation style:** manual annotation of every 2nd slice

**Description:** this is a label for the lateral ventricles, third and fourth ventricle. It is important to include the pellucid cyst, which is often seen in fetuses, sometimes visible in-between the dilated septum pellucidi (**Figure 7**). The frontal, occipital and temporal horns appear small in fetuses, however, if visible, it has to be annotated. It is important to pay attention to the non-intraventricular CSF spaces, such as the cisterns surrounding the midbrain – these have to be excluded from this label map.

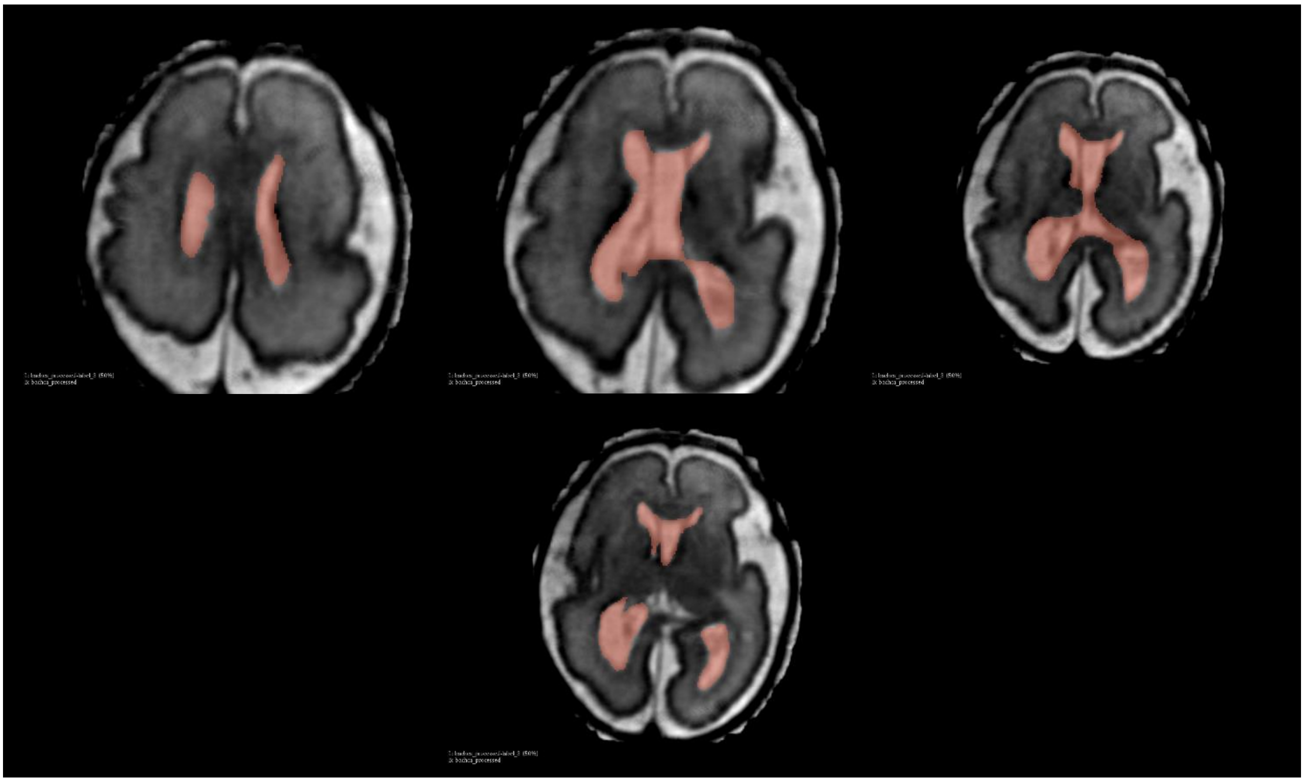

***Figure 7:*** *Label 4: annotating the lateral ventricles.*

The third ventricle (sometimes barely visible, thin CSF segment between the thalami) should be separated from the CSF spaces not belonging to the lateral ventricle, as shown in Figure 8:

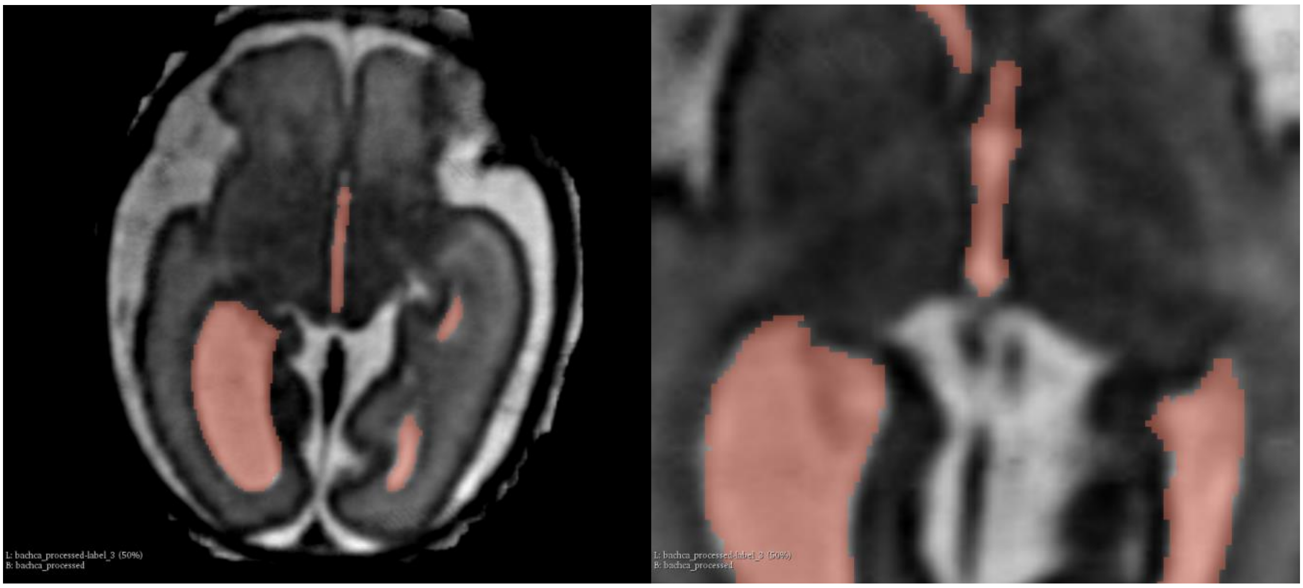


***Figure 8:*** *Label 4: annotating the third ventricle (part of label 4).*

Please note that the cisterns are NOT part of the lateral or third ventricles (**Figure 9**).

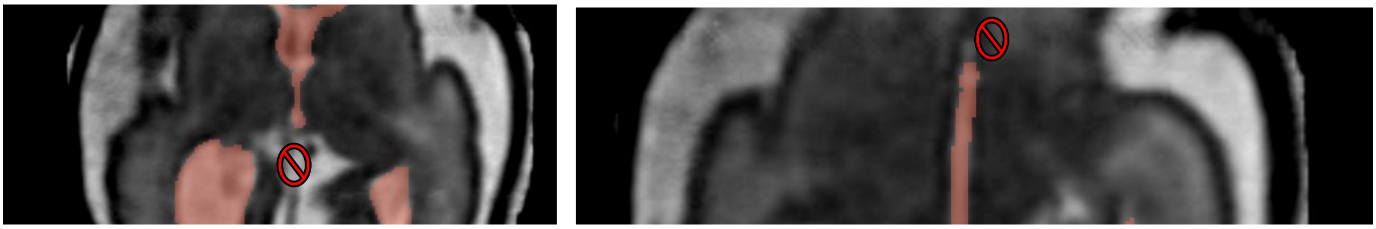

***Figure 9:*** *The cisterns surrounding the mesencephalon and brainstem should NOT be included in label 4. Similarly, the CSF space anterior to the AP, between the two hemispheres DO NOT belong to the ventricle annotation.*

The fourth ventricle is often seen, although only its more superior parts (**Figure 10**, arrow).

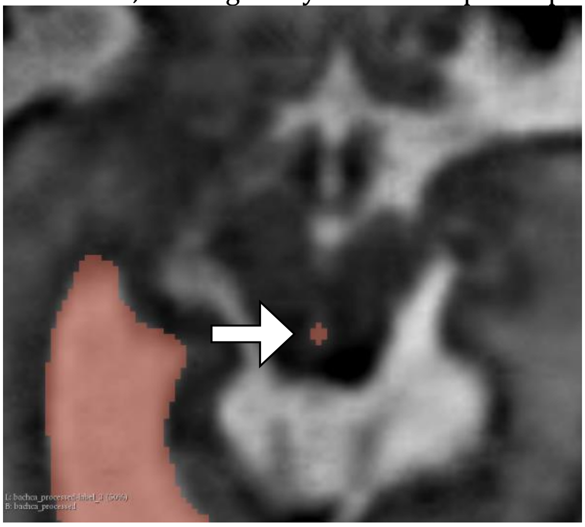

***Figure 10:*** *Label 4: annotating the fourth ventricle (part of label 4).*

## <u>Cerebellum</u> (label: 5)

**Annotation plane:** sagittal
**Annotation style:** manual annotation every 2nd slice

**Description:** this is a label for the cerebellum and the pedunculus cerebelli, not including the fourth ventricle. Please make sure that the fourth ventricle is excluded and the “>” shape is delineated mid-sagittally. The cerebellum is well visible on sagittal images as shown in **Figure 11**.

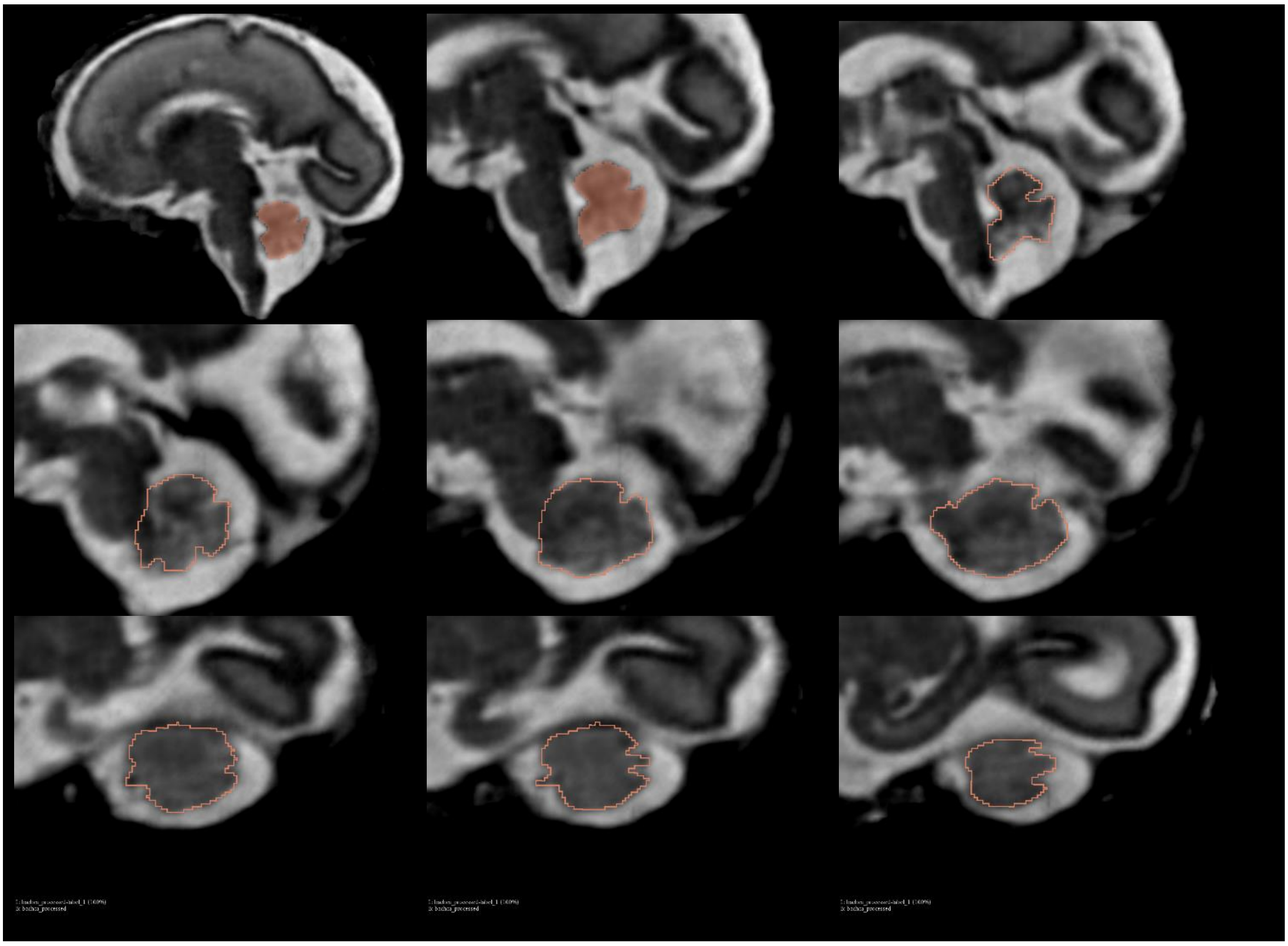

***Figure 11.*** *Label 5: annotation of the cerebellum, sagittal plane. Whenever possible, the cerebellar surface pattern has to be followed (foliae cerebri). Do not include the tentorium near the superior border of the cerebellar hemispheres.*

Make sure you check on coronal and axial images as well, to see if the brainstem is separated correctly from the cerebellum, as shown in **Figure 12**.

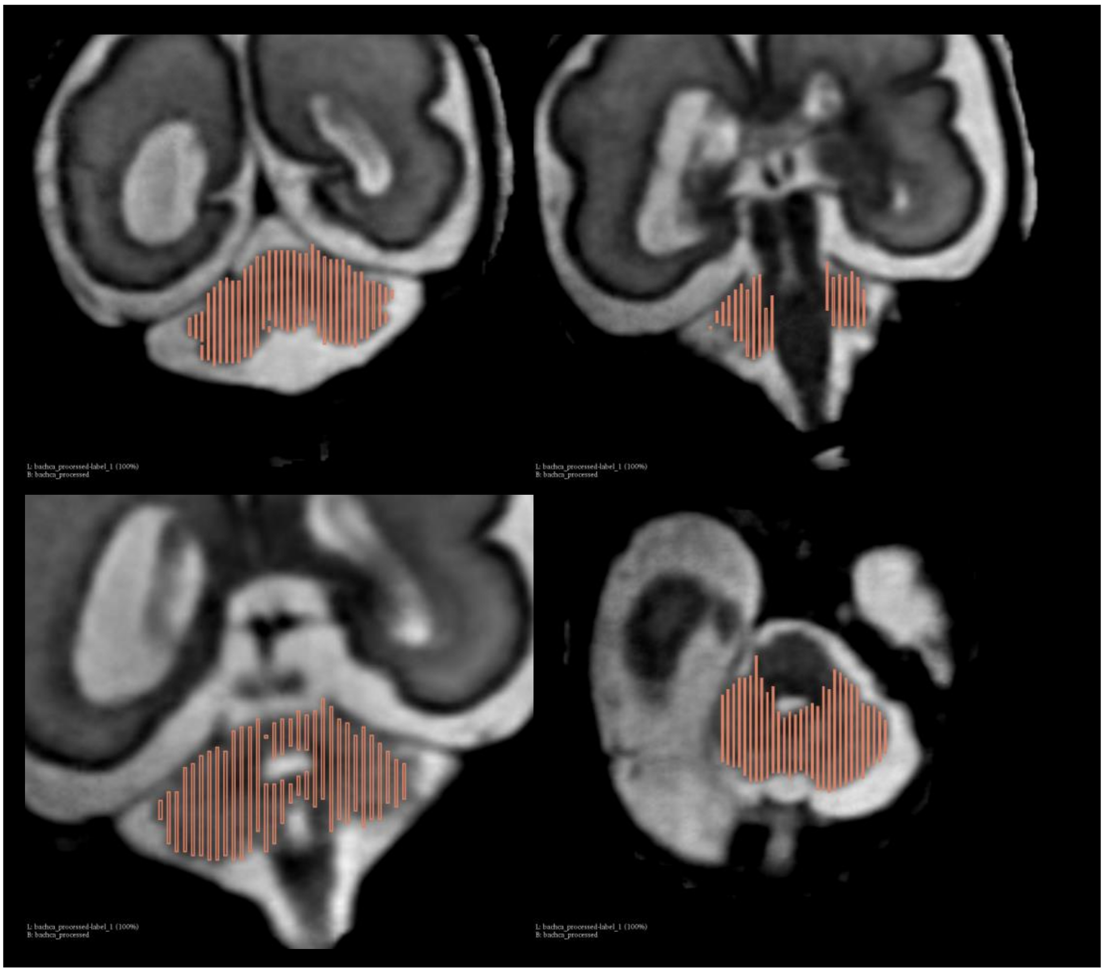

***Figure 12.*** *Label 5: annotation of the cerebellum, coronal plane. Please note the separation from the brainstem and pedunculus cerebelli.*

## <u>Deep gray matter</u> (label: 6)

**Annotation plane:** first axial, then coronal
**Annotation style:** manual annotation on a few (5-10) key slices in the axial plane (**Figure 13**) and then annotation in the coronal plane (**Figure 14**).

**Description:** this is a label for all the deep gray matter structures that should not belong to the white matter label. More precisely, this incorporates: thalamus, nucleus caudatus, globus pallidus, putamen, germinal matrix, and the hypothalamus. Basically, it is every non-WM structure that is surrounded by the external capsule. It does not include the colliculi, as that is part of the brainstem annotation. The internal capsule is within this label, as it is often not possible to separate due to low contrast in the 3D reconstructed T2 images.

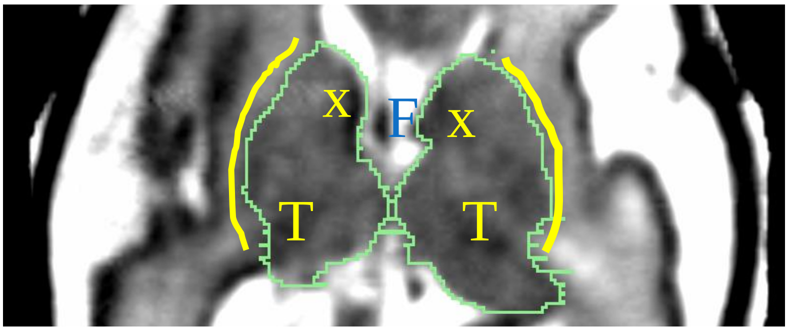


***Figure 13.*** *Label 6: annotation of the deep gray matter: key anatomical structures to include and avoid on the key axial slices, yellow line: external capsule, F: fornix, X: nucleus caudatus and germinal matrix, T: thalamus / globus pallidus (not separable due to low contrast).*

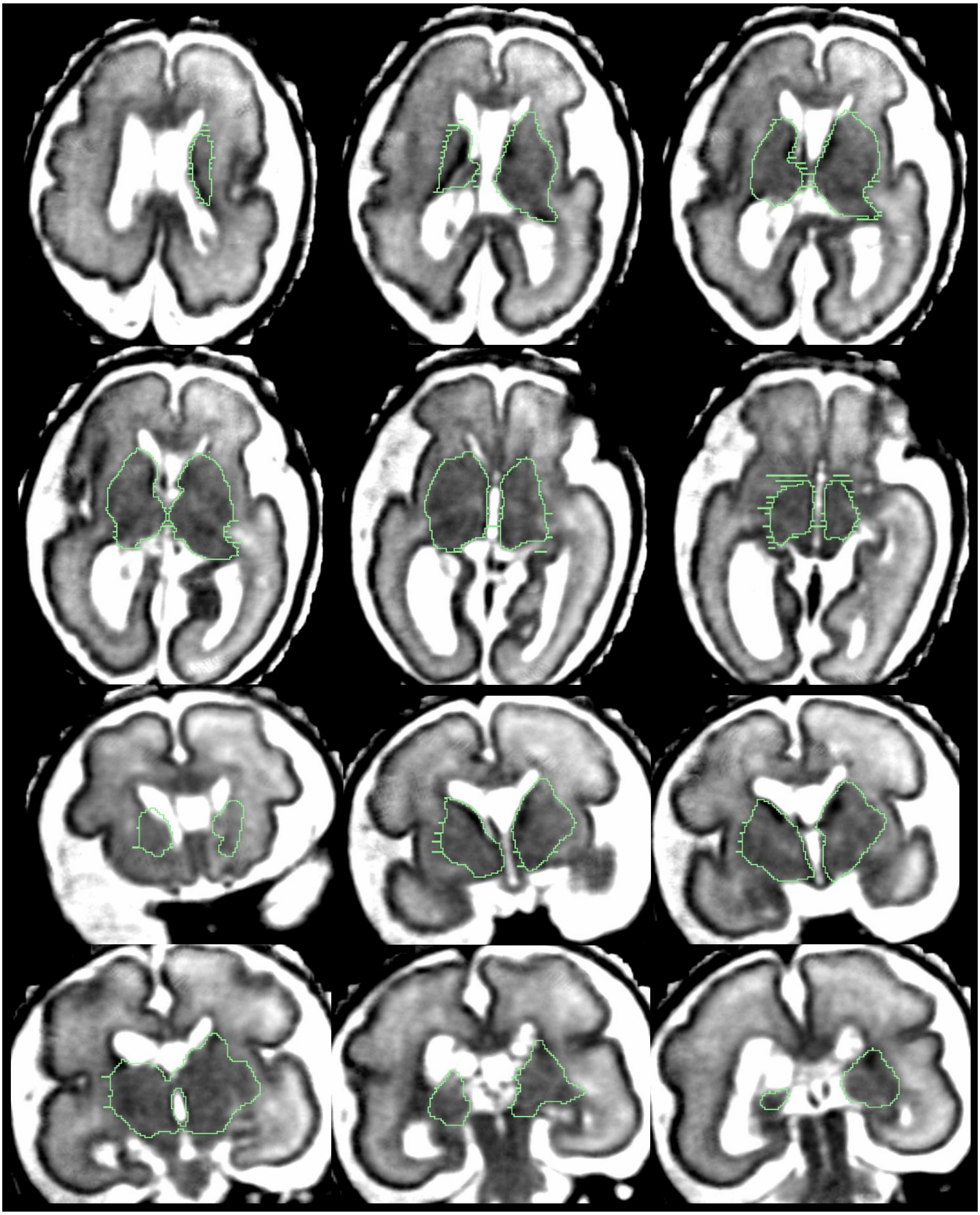

***Figure 14.*** *Label 6: annotation of the deep gray matter. The images show the label map after it has been annotated in both axial and coronal planes. First two rows: key axial slices to find the lateral extent of the deep gray matter*

## <u>Brainstem</u> (label: 7)

**Annotation plane:** first axial, then sagittal
**Annotation style:** manual annotation on a few key slices in the axial plane after locating the boundaries of the brainstem on the mid-sagittal images (**Figure 15**).

**Description:** this is a label for the spinal cord, brainstem, pons and the mesencephalon until the pedunculus cerebri. The label map also includes the hypophysis and the colliculi, which are important to be localized on sagittal images. In the axial plane, the L-R extent of the brainstem should first be delineated in slices across the mesencephalon and across many levels of the brainstem.

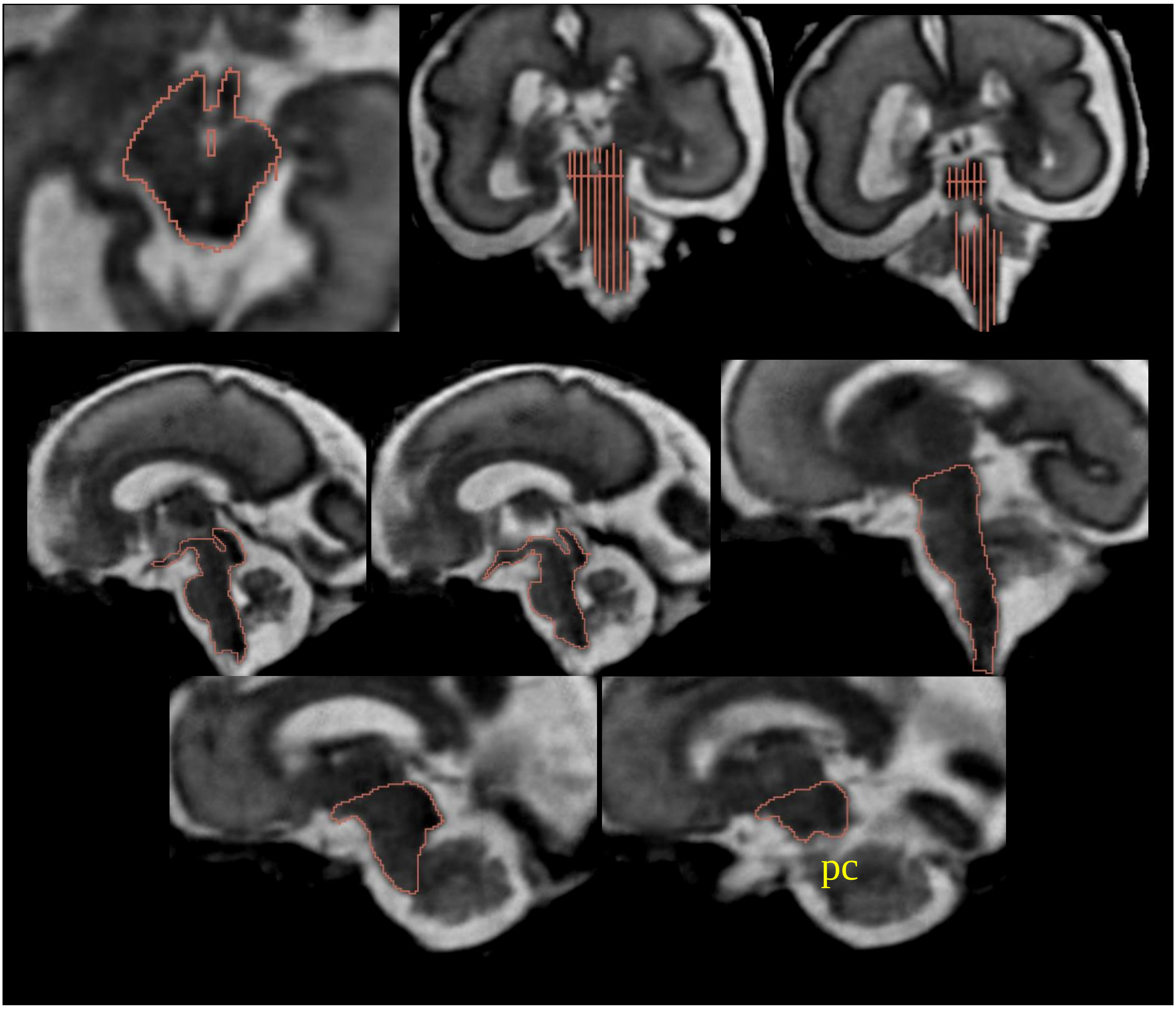


***Figure 15.** Label 7: annotation of the brainstem, sagittal plane. Please note the separation from the diencephalon (thalamus) and pedunculus cerebelli (marked with "pc").*

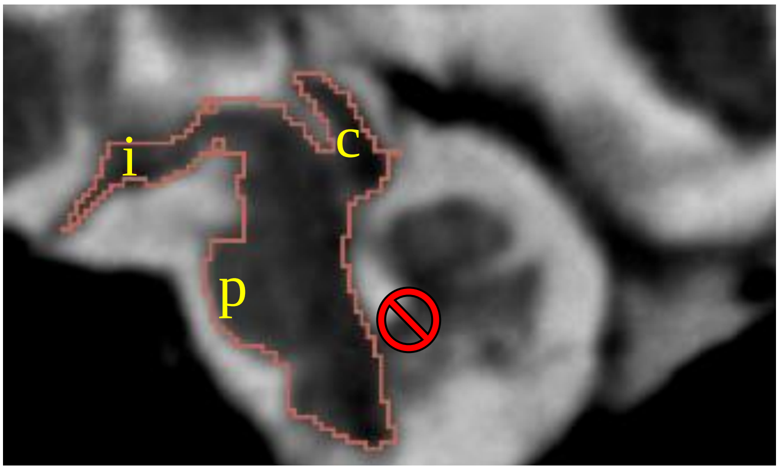


***Figure 16.*** *Label 7: annotation of the brainstem, sagittal plane. Important structures to be included: infundibulum of the hypophysis (i), colliculi ( c), pons (P), and the border to the cerebellum and fourth ventricle has to be carefully avoided (no-sign).*

The superior border of the brainstem has to be below the thalamus, optimally separated by a diagonal line as shown in **Figure 17**.

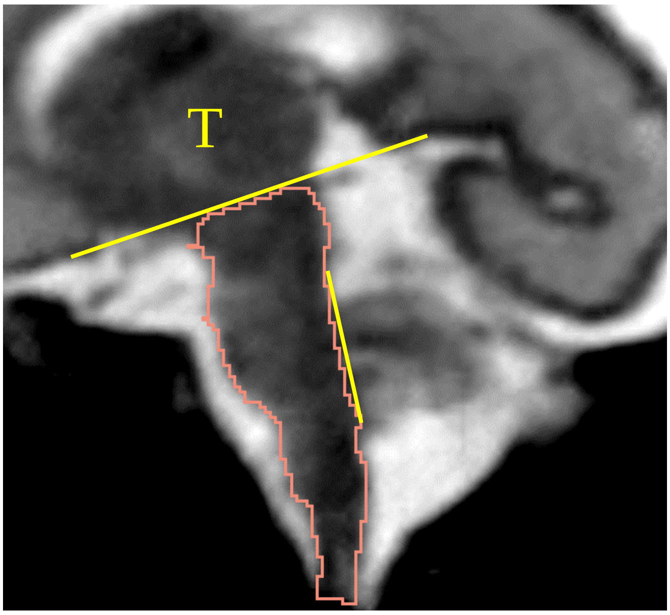


***Figure 17.*** *Label 7: annotation of the brainstem, sagittal plane. The brainstem can be delineated from the thalamus by an oblique plane, which should first be identified on the midsagittal image. Similarly, the pedunculi cerebelli / cerebellum has to be detached from this label with an oblique plane. T: thalamus*

Example annotations are provided in Annex 1.

**<u>Post processing of manually annotated label maps</u>**

As all label maps as only annotated on every 2nd – 3rd slices, it is necessary to interpolate along the direction which has been sparsely annotated. We also defined a given order of how label values overwrite each other if they are overlapping: **1 < 2 < 3 < 4 < 5 < 6 < 7**

We used an image processing script for minimal label map interpolation, smoothing and combination into the final label map. The code is distributed with the dataset.

**Annex 1:** Examples of completely annotated data sets

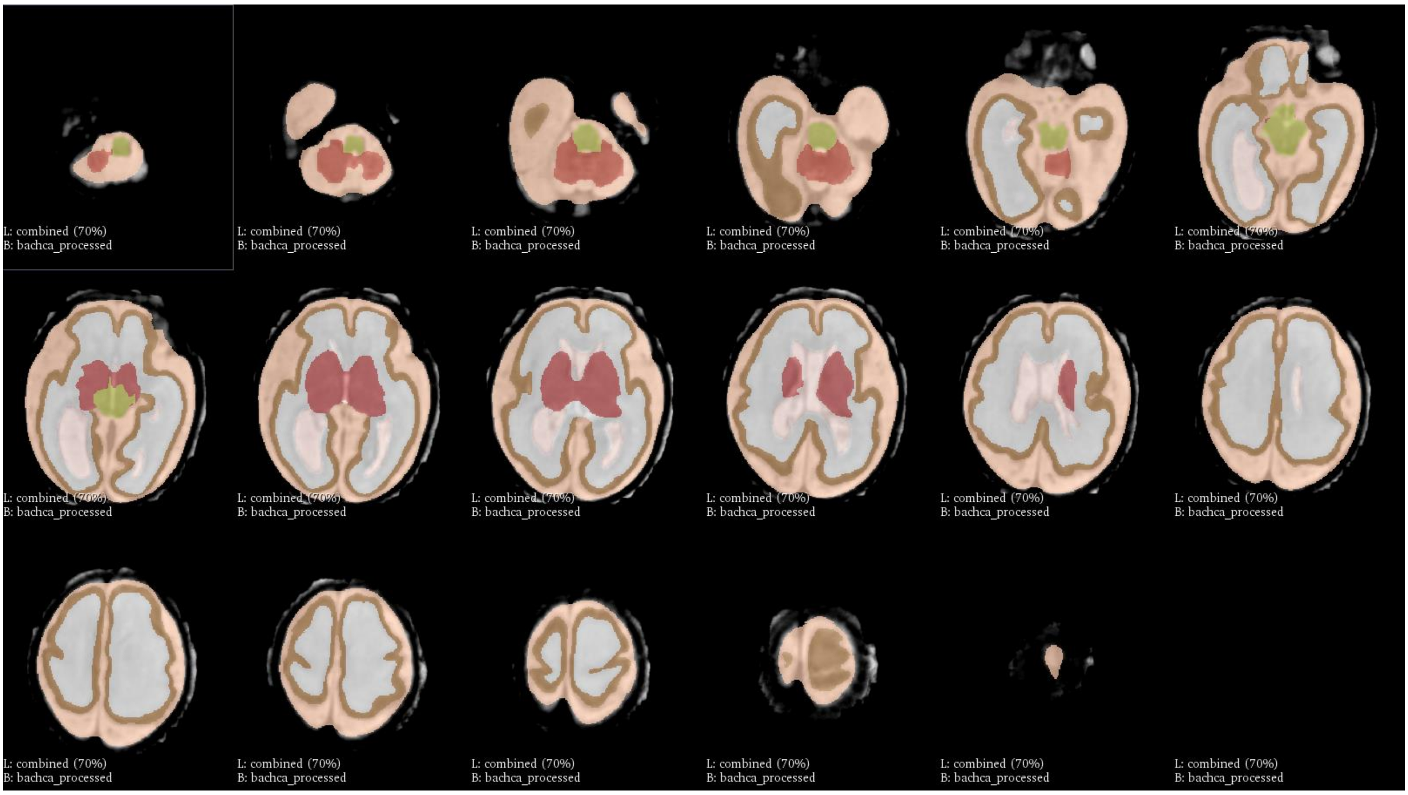


*Fetus, ca. 28. week of gestation, no brain abnormalities, good image quality, completely annotated fetal dataset after post processing and combination of manually drawn label maps. Please note that the non-default colours have been used in slicer for better visibility.*
